# An Impedimetric Biosensor Based on Ionic Liquid-Modified Graphite Electrodes Developed for microRNA-34a Detection

**DOI:** 10.3390/s18092868

**Published:** 2018-08-31

**Authors:** Ece Kesici, Ece Eksin, Arzum Erdem

**Affiliations:** 1 Analytical Chemistry Department, Faculty of Pharmacy, Ege University, Bornova 35100, Turkey; e.kesici93@gmail.com (E.K.); eceksin@hotmail.com (E.E.); 2 Biotechnology Department, The Institute of Natural and Applied Sciences, Ege University, Bornova 35100, Turkey

**Keywords:** ionic liquid, pencil graphite electrode, microRNA, electrochemical impedance spectroscopy, fetal bovine serum, nucleic acid hybridization

## Abstract

In the present work, an impedimetric nucleic acid biosensor has been designed for the purpose of detection of microRNA (miRNA). Ionic liquid (1-butyl-3-methylimidazolium hexafluorophosphate (IL))-modified chemically activated pencil graphite electrodes (PGEs) were used for the sensitive and selective detection of miRNA-34a. After covalent activation of the PGE surface using covalent agents (CAs), the ionic liquid (IL) was immobilized onto the surface of the chemically activated PGE by passive adsorption. The electrochemical and microscopic characterization of the IL/CA/PGEs was performed by electrochemical impedance spectroscopy (EIS), cyclic voltammetry (CV) and scanning electron microscopy (SEM). DNA probe concentration, miRNA target concentration, and also the hybridization time and wet adsorption time were optimized by using the EIS technique. Then, the hybridization occurred between specific DNA probes and miRNA-34a was immobilized onto the surface of the IL/CA/PGEs. The impedimetric detection of miRNA-DNA hybrid was performed by EIS. The detection limit (DL) was calculated in a linear concentration range of 2–10 µg/mL miRNA-34a target, and it was found to be 0.772 µg/mL (109 nM) in phosphate buffer solution (PBS) and 0.826 µg/mL (117 nM) in diluted fetal bovine serum (FBS). The selectivity of impedimetric biosensor for miRNA-34a was also tested against to other non-complementary miRNA sequences both in buffer media, or diluted FBS.

## 1. Introduction

Nucleic acid analysis using biosensing strategies has been an attractive topic in many fields, including gene analysis, clinical disease diagnosis, biological, environmental, pharmaceutical and forensic applications [1,2,3,4,5,6]. Nucleic acid recognition technologies combined with electrochemical transducers have been applied to the diagnostic area. Wide-scale genetic testing requires the development of easy-to-use, fast, inexpensive, miniaturized analytical devices.

Ionic liquids (ILs) like 1-butyl-3-methylimidazoliumhexafluorophosphate are known as low-melting organic salts that are mostly liquid at room temperature. Due to their unique properties such as low measurable vapor pressure, high thermal stability and conductivity, good solvating properties, non-volatility, low toxicity and biocompatibility, they have been used in different fields, including development of electrochemical biosensors [7,8,9,10,11,12,13,14,15]. Ren et al. [15] developed a chronocoulometric DNA sensor that based on polyaniline nanotubes (PANINTs) and ionic liquid (IL)-doped screen-printed electrodes. Eksin et al. [13] developed chitosan/ionic liquid modified pencil graphite electrodes (CHIT/IL/PGEs) in order to perform enhanced electrochemical monitoring of nucleic acid, and study the interaction of the anticancer drug, mitomycin C (MC) and calf thymus double stranded DNA (dsDNA). She et al. [14] detected hydroquinone using a carbon paste electrode enhanced by a hydrophobic IL. Sengiz et al. [16] designed IL modified pencil graphite electrodes (IL/PGEs) for electrochemical monitoring of DNA sequence selective hybridization related to *Microcystis* spp. (MYC).

miRNAs are small nucleotides in 19–25 bp. that are noncoding RNA molecules found in eukaryotic cells [17,18]. The importance of miRNA itself is due to the complicated regulatory functions it plays in various life processes and its close relationship with some diseases. The majority of diseases (cancer, heart failure, vascular disease, diabetes, etc.) has been related to the regulation of miRNAs due to their influence on the fundamental cellular process including cell proliferation, apoptosis, differentiation, and migration [2,19,20]. miRNA-34a in particular is related to cancer initiation, oncogenesis, and tumor response to treatments [21]. According to the literature, it was found that miRNA-34a could be used as biomarker for diagnosis of cancer [22,23], cardiovascular disease [24] and Alzheimer’s disease [25].

Northern blotting, isothermal exponential amplification-based methods and rolling cycle amplification-based methods are applied as the conventional miRNA detection techniques which are expensive, complicated, hardly employed and time consuming for on-site measurement. In contrast to these conventional techniques, the electrochemical detection techniques in combination with biosensor technologies have more advantages such as time savings, simple analysis by offering a specific recognition process and requiring a small amount of sample. Efficient immobilization of oligonucleotides onto the surface of the analysis platform has been one of the key features for the successful design of biosensor platforms [1,2,3,4,5,6,26]. Electrochemical techniques offer a reliable and fast response to achieve sensitive and selective detection and to miniaturize the fabricated biosensors. Electrochemical impedance spectroscopy (EIS) is one of the sensitive analytical techniques that can rapidly detect small changes in reactive surfaces by using a transducer and enlarging the signal with an amplifier, making EIS one of the more suitable detection methods for nucleic acid biosensors [27]. Additionally, EIS allows one to perform analyses in complex solutions, such as serum.

To the best of our knowledge, this is the first study in the literature which presents the impedimetric detection of miRNA-34a by using ionic liquid-modified chemically activated PGEs. IL/CA/PGEs are designed for the purpose of specific recognition of miRNA-34a, which is related to Alzheimer’s disease [25,28] and different types of cancer [22,23,29,30]. 

First, the chemical activation of the PGE surface was performed by covalent agents in order to allow the efficient modification of the electrode surface with the IL due to its advantages; such as, its enhanced conductivity due to its stability and making sensor’s surface biocompatible. The characterization of the IL-CA-modified PGEs was performed by scanning electron microscopy (SEM), electrochemical impedance spectroscopy (EIS) and cyclic voltammetry (CV). The experimental parameters, such as duration of the IL modification on the surface of the PGE, the percentage of IL, miRNA-34a specific DNA probe, miRNA-34a RNA concentration, the hybridization time and hybrid immobilization time onto the surface of IL/CA/PGEs were optimized. The detection limit (DL) of miRNA-34a was estimated, and the selectivity of the impedimetric biosensor platform was tested against to other non-complementary microRNA sequences; miRNA-155 and miRNA-181b in buffer (PBS, pH 7.40), or fetal bovine serum (FBS):PBS (1:10) diluted solution.

## 2. Materials and Methods

### 2.1. Apparatus

The electrochemical measurements including CV and EIS were carried out using an AUTOLAB PGSTAT 302 electrochemical analysis system equipped with the GPES 4.9 software package (Eco Chemie, Utrecht, The Netherlands).

The measurements were performed with a traditional three electrode system using a disposable PGE, a platinum wire and an Ag/AgCl/KCl/3M (BAS, Model RE-5B, W. Lafayette, IN, USA) as working, counter and reference electrode, respectively. All measurements were performed in a Faraday cage (Eco Chemie, Utrecht, The Netherlands). A model pencil (Rotring, Hamburg, Germany) was used as a holder for the graphite lead (Tombow 0.5 HB, Shinshiro, Japan). Electrical contact between the lead and pencil was provided by soldering a metallic wire to the metallic part of the pencil. The pencil lead was held vertically with 14 mm of the lead extruded outside and 10 mm of which was immersed in the solution.

### 2.2. Chemicals

The chemical linkers used for covalent attachment; *N*-hydroxysuccinimide (NHS) and *N*-(3-dimethylaminopropyl)-*N*′-ethylcarbodiimide hydrochloride (EDC) were purchased from (Sigma-Aldrich, St. Louis, MO, USA). The IL (1-butyl-3-methylimidazolium hexafluorophosphate) was also purchased from Sigma-Aldrich. Other chemicals were in analytical reagent grade and supplied by Sigma (St. Louis, MO, USA) and Merck (Kenilworth, NJ, USA).

The amino linked miRNA-34a specific DNA probe, miRNA-34a RNA target (i.e., the complementary of miRNA-34a specific DNA probe), and other miRNA sequences were purchased from Ella Biotech (Planegg, Germany) as a lyophilized powder. The base sequences of the miRNAs are listed below:

**miRNA-34a G specific DNA probe: (miRNA-34a G probe):**

5′-NH_2_-(CH_2_)_6_-ACA ACC AGC TAA GAC ACT GCC A-3′

**miRNA-34a RNA target:**

5′-UGG CAG UGU CUU AGC UGG UUG U-3′ (U= Urasil)

**Non-complementary (NC) miRNAs:**

miRNA-155:

5′-UUA AUG CUA AUC GUG AUA GGG GU-3′

miRNA-181b:

5′-AAC AUU CAU UGC UGU CGG UGG GU-3′

The stock solutions of miRNAs (500 μg/mL) were prepared with Tris–EDTA buffer (10 mM Tris–HCl, 1 mM EDTA, pH 8.0) and kept frozen. The diluted solutions of miRNAs were prepared with 50 mM phosphate buffer containing 20 mM NaCl (PBS, pH 7.4).The CA solution was prepared freshly for each experiment by using 5 mM EDC and 5 mM NHS prepared in phosphate buffer solution (PBS, pH 7.40). Ultra-pure water was used in order to prepare all aqueous solutions.

### 2.3. Procedure

The experimental procedure consists of following steps:

(i) pretreatment of PGE (+1.4 V, 30 s)
(ii) chemical activation of PGE surface using covalent agents (CA)
(iii) IL modification onto CA/PGE surfaces
(iv) characterization of IL/CA/PGEs
(v) hybridization of miRNA-34a specific DNA probe with miRNA-34a RNA target, or non-complementary RNAs (miRNA-155/miRNA-181b)
(vi) immobilization of hybrid onto the surface of IL/CA modified electrode
(vii) electrochemical measurement

**Figure 1:**
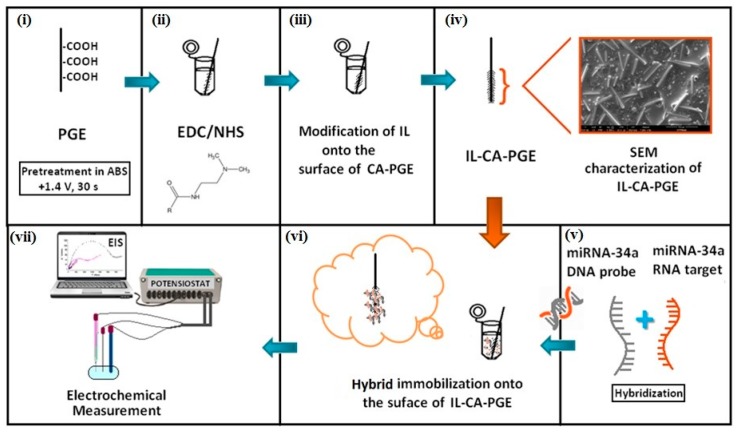
Experimental scheme presenting the pretreatment of PGE (**i**); the activation of PGE with EDC/NHS (**ii**), modification of IL onto the surface of CA/PGE (**iii**); microscobic characterization of electrodes (**iv**), the hybridization between miRNA-34a target and its complementary DNA probe (**v**); immobilization of the hybrids at the surface of IL/CA/PGE (**vi**); electrochemical measurement (**vii**).

PGE surface activation processes were carried out at +4 °C. Other experiments were done at room temperature. A schematic representation was presented in Figure 1.

#### 2.3.1. Preparation of CA Solution and Formation of Chemically Activated PGEs

PGEs were electrochemically pretreated by applying +1.40 V for 30 s in acetate buffer solution (ABS, pH 4.80) (Figure 1i) [6,7,13,31,32]. Each pretreated PGE was immersed into the vials containing 100 μL of 5 mM CA solution during 60 min to obtain activated carboxyl groups at the surface of the PGEs [31] before IL modification. Each PGEs was then rinsed with PBS for 10 s to remove unbound probe from electrode surface (Figure 1ii). NHS is an organic compound and after its mixing with a coupling reagent such as EDC, activated acid intermediate (CA) could be formed.

#### 2.3.2. Modification of IL onto the Surface of CA/PGEs

The required amount of IL dissolved in organic solvent *N*,*N*-dimethylformamide (DMF) solution and then sonicated during 30 min at room temperature. According to the experimental conditions for preparation of IL modified electrodes previously reported by our group, each chemically activated PGE was immersed into the vials containing 100 µL of IL during 15 min (Figure 1iii). Then, the electrodes were then allowed to dry for 30 min at upside position without rinsing [16].

#### 2.3.3. Microscopic Characterization of the Electrodes by Scanning Electron Microscopy (SEM)

The SEM images of unmodified, CA modified, IL modified, and CA and IL modified PGEs were obtained by a Quanta 400 FEI (Thermo Fisher Scientific, Hillsboro, OR, USA), field emission scanning electron microscope (FE-SEM, Tokoyo, Japan) with the acceleration voltage 5.0 kV with 5, 10 and 50 µm resolutions.

#### 2.3.4. Hybridization of miRNA-34a Specific DNA Probe with miRNA-34a Target or Non-Complementary miRNAs

The miRNA-34a specific DNA probe, or miRNA-34a target or non-complementary miRNAs; miRNA-155/miRNA-181b was prepared in PBS (pH 7.40). Accordingly, the DNA probe and miRNAs were mixed and incubated at room temperature with gently mixing at 400 rpm during 5 min for hybrid formation in solution phase (Figure 1v). Likewise, the hybridization of miRNA-34a specific DNA probe and complementary miRNA-34a RNA target, or non-complementary miRNAs was also performed in the artificial serum medium; e.g., FBS medium during 5 min at room temperature.

#### 2.3.5. Immobilization of DNA-RNA Hybrids onto the Surface of IL/CA/PGEs

The chemically active and IL modified PGE was immersed into the vials containing 40 µL hybrid during 30 min in order to immobilization of hybrid onto the electrode surface according to wet-adsorption process (Figure 1vi). Each of the electrodes was then rinsed with PBS for 10 s to remove unbound probe from electrode surface, and accordingly the impedimetric measurements were performed.

#### 2.3.6. Cyclic Voltammetry Measurements

CV measurements were performed in potential range from −0.5 to +1.30 V with the scan rate as 50 mV/s in a redox probe solution of 2 mM K_3_[Fe(CN)_6_]/K_4_[Fe(CN)_6_] (1:1) prepared in 0.1 M KCl. The raw datas were also treated using the Savitzky and Golay filter (level 2).

#### 2.3.7. Impedimetric Measurements

In the presence of 2.5 mM K_3_[Fe(CN)_6_]/K_4_[Fe(CN)_6_] (1:1) mixture as redox probe prepared in 0.1 M KCl, EIS measurements were performed accordingly in each experimental step (Figure 1vii). At the open circuit potential of +0.23 V versus Ag/AgCl with a sinusoidal signal of 10 mV, the impedance was measured in a frequency range from 10 mHz to 1000 kHz. The respective semicircle diameter corresponds to the charge transfer resistance (R_ct_), the values are calculated using the fitting program AUTOLAB PGSTAT 302 (FRA version 4.9, Eco Chemie, Utrecht, The Netherlands).

All measurements are performed in triplicate by using new electrode and the relative standard deviations (RSD%) indicate reproducibility of the results.

## 3. Results and Discussion

**Figure 2:**
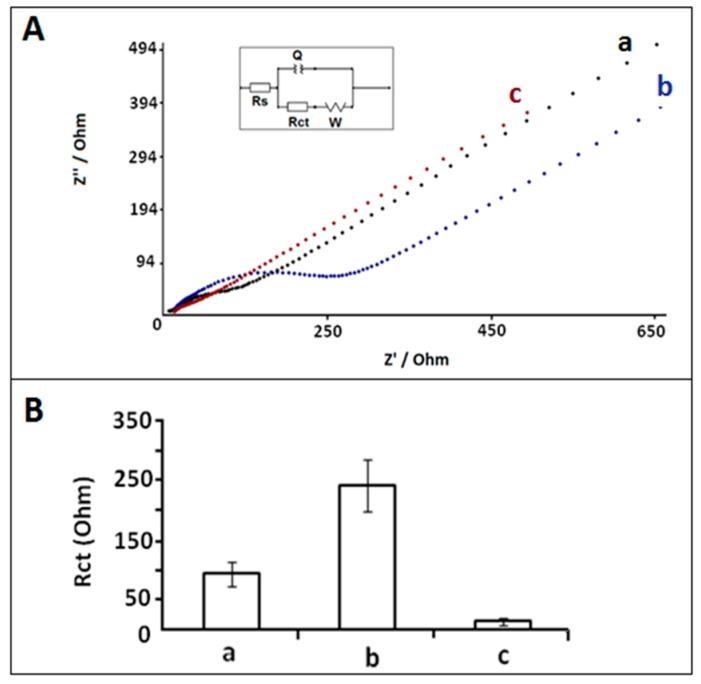
(**A**) Nyquist diagrams of (**a**) PGE, (**b**) CA/PGE, (**c**) IL/CA/PGE in 2.5 mM Fe(CN)_6_^3−/4−^. (**B**) Histograms representing the average R_ct_ values of (**a**) PGE, (**b**) CA/PGE, (**c**) IL/CA/PGE (n = 3). Inset was the equivalent electrical model used to fit the impedance data. The impedimetric results were fitted by using an equivalent circuit model, R(Q(RW)) the parameters of which are listed in the text; R_S_ is the solution resistance. The constant phase element Q is then related to the double layer capacitance at the electrode-electrolyte interface. R_ct_ is related to the charge transfer resistance at the electrode-electrolyte interface. The constant phase element W is the Warburg impedance due to mass transfer to the electrode surface.

The electrochemical surface characterization was investigated by using CV and EIS. The impedimetric surface characterization of PGE, CA/PGE and %20 IL modified CA/PGE were performed by EIS technique in order to introduce the advantages of CA activation and IL modification onto PGE surface (Figure 2). The average R_ct_ values of unmodified PGE, CA/PGE, and IL/CA/PGE were measured as 103.26 ± 23.14 Ohm (RSD%, 22.41%, n = 3), 254.00 ± 49.56 Ohm (RSD%, 19.51%, n = 3) and 15.67 ± 3.07 (RSD%, 19.58%, n = 3). After IL modification onto CA/PGE surface, 93.83% decrease at R_ct_ was recorded (Figure 2b,c). This decrease is consistent with the fact that the IL modification can yield an increase at the conductivity of PGE surface since there is an increase at the interfacial electron transfer occurring between the electrode and the electroactive species in solution [31].

**Figure 3:**
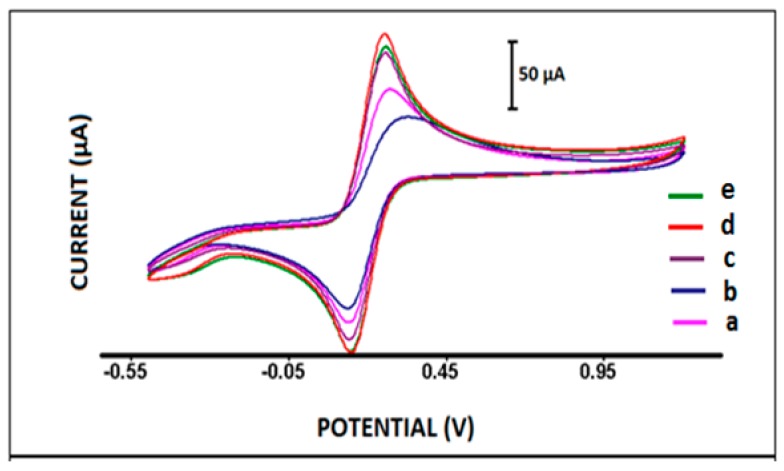
Cyclic voltammograms recorded in 2 mM K_4_[Fe(CN)_6_ ]/K_3_[Fe(CN)_6_] (1:1) prepared in 0.1 M KCl solution by using (**a**) PGE and (**b**) CA/PGE, %IL modified CA/PGE (**c**) 10% (**d**) 20% (**e**) 30%.

Next, the electrochemical surface characterization of PGE, CA/PGE and IL/CA/PGE was determined by using CV as seen in Figure 3. The average anodic peak current (Ia) of PGE, CA/PGE and 20% IL modified CA/PGE was measured as 81.95 ± 4.33 µA (RSD%, 5.29%, n = 3), 60.07 ± 2.68 µA (RSD%, 4.46%, n = 3) and 122.33 ± 10.64 µA (RSD%, 8.70%, n = 3) Figure 3. After IL modification onto CA/PGE surface 103.60% increase at anodic peak current was recorded (Figure 3b–d) due to the fact that the repulsive electrostatic interaction between IL and anionic redox [Fe(CN)6]^3−/4−^ ions at the surface of IL/CA/PGE. The role of IL is to accelerate the electron transfer and accordingly, an increase at Ia was recorded comparison to the ones obtained by PGE and CA/PGE.

In order to the see the effect of IL concentration upon the average anodic peak current (Ia) to was examined in various IL concentrations; 5%, 10%, 15%, 20%, 25% and 30% and the results shown in Figure S1. The average anodic peak currents (Ia) of 5%, 10%, 15%, 20%, 25% and 30% was measured as 112.10 ± 4.60 µA (RSD%, 4.11%, n = 3), 116.10 ± 4.45 µA (RSD%, 3.83%, n = 3), 122.33 ± 10.64 µA (RSD%, 8.70%, n = 3), 115.10 ± 7.00 µA (RSD%, 6.07%, n = 3) and 116.23 ± 5.37 µA (RSD%, 4.62%, n = 3). The maximum increase in anodic peak current was observed at 20% IL concentration. According to the CV measurements; average anodic peak currents (Ia), anodic charge values (Qa) and calculated surface areas (A) of PGE, CA/PGE and % IL modified CA/PGEs, 5% IL to 30% IL are shown in Table S1. 20% IL modified CA/PGE was chosen as optimum IL concentration due to the maximum surface area was determined about 50%. Moreover, the largest surface area as 0.38 cm^2^ was obtained by IL/CA/PGE in contrast to the ones of PGE and CA/PGE. Hence, it was concluded that the electroactive surface area of CA/IL/PGE increased due to the layered structure of IL in comparison to the one of CA/PGE.

In addition, IL immobilization period onto the surface of CA/PGE was optimized by EIS in Figure S2. The average R_ct_ values of unmodified PGE, CA/PGE, and IL modified CA/PGE in 15, 30 and 60 min were measured as 103.94 ± 22.44 Ohm (RSD%, 21.59%, n = 3), 251.06 ± 45.51 Ohm (RSD%, 18.13%, n = 3), 19.67 ± 4.04 (RSD%, 20.55%, n = 3), 19.79 ± 6.20 (RSD%, 31, 32%, n = 3) and 21.00 ± 8.19 (RSD%, 38.98%, n = 3) respectively.

The highest decrease at the R_ct_ value was obtained in the case of 15 min passive adsorption and it was more reproducible. Also, all detection time can be shorter here with 15 min passive adsorption time was used in further experiments.

In addition, the apparent fractional coverage (Q^IS^_R_) values were calculated according to Equation (1) given by earlier report of Janek et al. [32] to describe the surface to which the probe was attached to the surface to the surfaces of PGE, CA/PGE and IL/CA/PGE:

Q^IS^_R_ = 1 − [R_ct_ (bare electrode)/R_ct_ (modified electrode)] (1)

The results are summarized in the inset of Figure S3. The highest Q^IS^_R_ was found in the presence of 1 μg/mL probe onto the surface of IL/CA/PGE.

**Figure 4:**
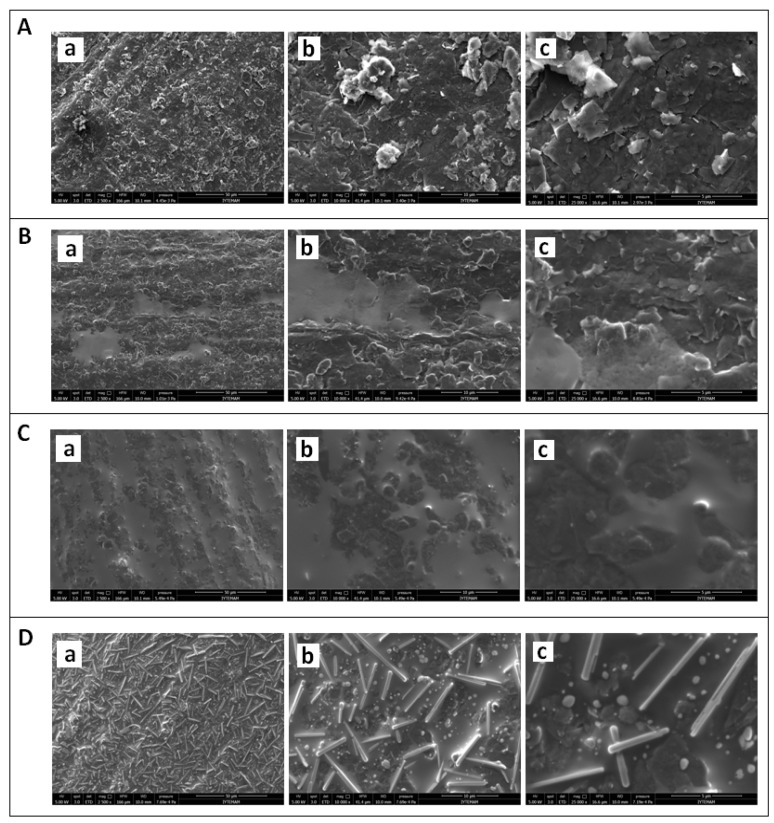
SEM images of (**A**) unmodified PGE, (**B**) CA modified PGE, (**C**) IL modified PGE, (**D**) CA and IL modified PGE using identical acceleration voltage as 5.0 kV with resolution at (**a**) 50 µm, (**b**) 10 µm and (**c**) 5 µm respectively.

In addition, the microscopic characterization of disposable graphite electrodes; unmodified PGE, CA/PGE, IL/PGE and IL/CA/PGEs was explored by SEM under the optimum conditions. The SEM images are given in Figure 4. After activation of the PGE surface by using CA agents, a brighter surface observed in contrast to the unmodified one due to the film layer at the CA modified electrode surface (Figure 4B). It was found that the surface of IL modified PGE is more homogenous than the one of unmodified PGE (Figure 4A).

After IL modification onto the surface of CA/PGE (Figure 4D), a more homogenous surface was obtained in contrast to the ones of CA/PGE (Figure 4B) and IL/PGE (Figure 4C).

In order to optimize the miRNA-34a DNA probe concentration, hybridization was performed in the presence of different miRNA-34a DNA probe concentrations and 10 µg/mL miRNA-34a target in Figure S4. The average R_ct_ values after pseudo-hybridization of 0.5, 1.0 and 1.5 µg/mL DNA probe were measured as 227.50 ± 4.95 Ohm; 238.67 ± 24.91 Ohm and 344.50 ± 45.96 Ohm, respectively. After full hybridization of 0.5, 1.0 and 1.5 µg/mL miRNA-34a DNA probe with 10 µg/mL miRNA-34a target, the average R_ct_ values were measured as 689.17 ± 83.44 Ohm; 815.00 ± 331.31 Ohm and 604.50 ± 101.12 Ohm, respectively.

In the case of full hybridization between 0.5 µg/mL DNA probe and 34a target, the R_ct_ value was found 2.88 times higher than the one obtained in the pseudo-hybridization. This increase at R_ct_ value was explained by the increased negativity at the electrode surface after DNA:miRNA hybridization. Furthermore, the highest increase at R_ct_ value was determined in the presence of full match hybridization case of 1 µg/mL miRNA-34a DNA probe with miRNA-34a target. Hence, 1 µg/mL was selected as optimum miRNA-34a probe concentration.

Next, the effect of hybridization time was explored based upon the sensor response. The hybridization between 1 µg/mL miRNA-34a DNA probe and 10 µg/mL miRNA34-a target was performed for various hybridization times (5, 15, 30 min, Figure S5). After 5 min pseudo-hybridization of 1 µg/mL miRNA-34a probe, the average R_ct_ value was measured as 277.71 ± 66.42 Ohm (RSD% = 23.92%, n = 3). After hybridization between 1 µg/mL miRNA-34a DNA probe and 10 µg/mL miRNA-34a target, the average R_ct_ value was 2.5 times higher than the one obtained by pseudo-hybridization 689.40 ± 95.95 Ohm (RSD% = 13.92%, n = 3) (Figure S5). The highest increase at the R_ct_ value was obtained in the case of 5 min hybridization, thus 5 min hybridization time was used in further experiments.

In addition, the effect of passive adsorption time of hybrid was investigated. The miRNA-34a DNA probe (1 µg/mL) and 10 µg/mL miRNA34-a target hybrid was immobilized onto the surface of IL/CA/PGE for various passive adsorption times (5, 15, 30, 45 min, Figure S6). After 30 min passive adsorption of 1 µg/mL miRNA-34a DNA probe and 10 µg/mL miRNA-34a target, the average R_ct_ value was measured as 691.80 ± 93.01 (RSD% = 13.44%, n = 3). This was 2.4 times higher than the one obtained by pseudo-hybridization 287.00 ± 78.02 Ohm (RSD% = 27.19%, n = 3, Figure S4). The highest increase at the R_ct_ value was obtained in the case of 30 min passive adsorption.

**Figure 5:**
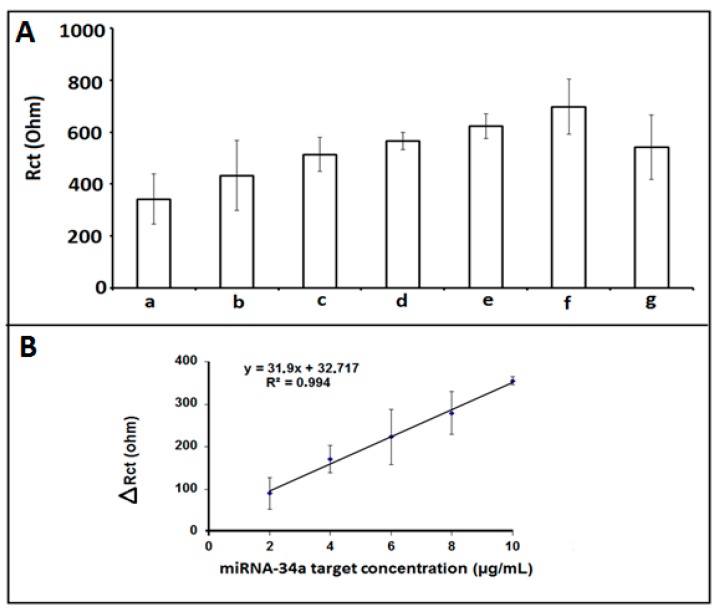
(**A**) Histograms representing the R_ct_ values obtained by (**a**) 1 µg/mL miRNA-34a DNA probe, after hybridization between 1 µg/mL miRNA-34a DNA probe and different miRNA-34a RNA target concentrations (**b**) 2 µg/mL, (**c**) 4 µg/mL, (**d**) 6 µg/mL, (**e**) 8 µg/mL, (**f**) 10 µg/mL (**g**) 12 µg/mL. (**B**) The calibration graph obtained from R_ct_ values measured in the presence of different concentration of miRNA-34a target, ranging 2–10 µg/mL.

The effect of miRNA-34a target concentration on the hybridization process based on the changes at R_ct_ value was investigated. After 5 min pseudo-hybridization of 1 µg/mL miRNA-34a DNA probe, the average R_ct_ value was found to be 342.27 ± 97.28 Ohm (RSD% = 28.42%, n = 3) in Figure 5. Then, the R_ct_ values were measured in the case of full hybridization between 1 µg/mL miRNA-34a DNA probe and miRNA-34a target at different concentrations varying from 2 to 12 µg/mL and the average R_ct_ values were measured 433.00 ± 134.54 Ohm; 513.50 ± 65.76 Ohm; 565.67 ± 32.58 Ohm; 622.00 ± 47.48 Ohm, 697.75 ± 106.30 Ohm; 542.00 ± 123.04 Ohm, respectively. The highest R_ct_ value with a good reproducibility (RSD% = 15.23%, n = 3) was determined in the presence of 10 µg/mL miRNA-34a target.

Moreover, the apparent fractional coverage values Q^IS^_R_ were calculated for hybridization between 1 µg/mL miRNA-34a probe and 10 µg/mL mirNA-34a DNA target on the surface of IL/CA/PGE according to Equation (1) described by Janek et al. [32] and found to be 0.955 (Table S2). The values of Q^IS^_R_ were higher than 0.9, which indicated successful modification and the more coverage was obtained at the surface of hybrid between 1 µg/mL miRNA-34a probe and 10 µg/ mL mirNA-34a DNA target.

The detection limit (DL) was calculated in the linear concentration range from 2 to 10 μg/mL (Figure 5B, inset) and it was found to be 0.772 μg/mL (4.36 pM in 40 μL sample) with a regression equation y = 31.9x + 32.717 with the coefficient of determination (R^2^) of R² = 0.994 according to the procedure described by Miller and Miller [33]. Since the highest and more reproducible R_ct_ was obtained in the presence of hybridization of 1 μg/mL miRNA-34a I probe with 10 μg/mL miRNA-34a target, the 10 μg/mL target concentration level was chosen as the optimum one for further selectivity studies.

**Figure 6:**
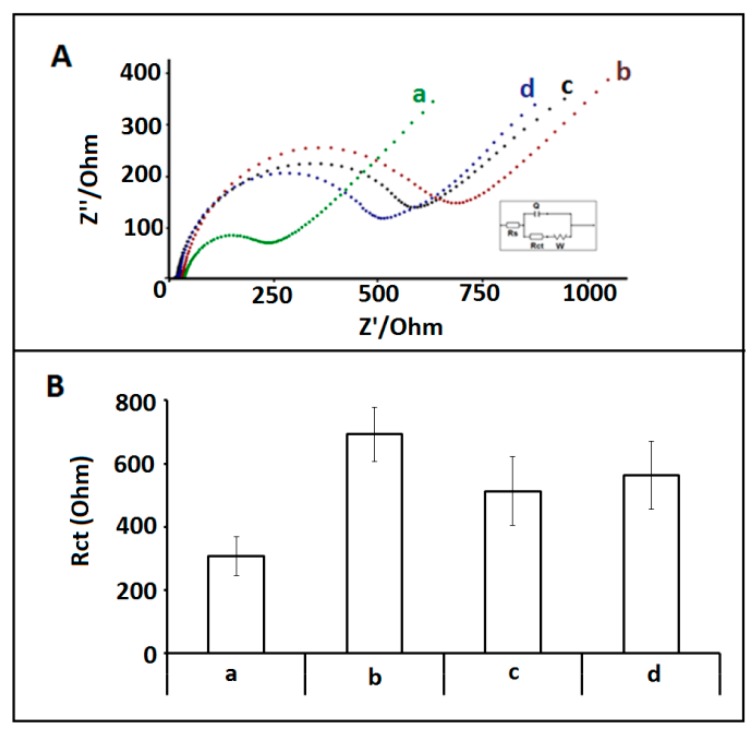
(**A**) Nyquist diagrams representing the R_ct_ values obtained by (**a**) 1µg/mL miRNA-34a probe modified /IL/CA/PGE, hybridization between 1 µg/mL miRNA-34a DNA probe and 10 µg/mL, (**b**) miRNA-34a target, (**c**) miRNA-181b, (**d**) miRNA-155. (**B**) Histograms representing the R_ct_ values obtained by (**a**) 1µg/mL miRNA-34a probe modified /IL/CA/PGE, hybridization between 1 µg/mL miRNA-34a DNA probe and 10 µg/mL, (**b**) miRNA-34a target, (**c**) miRNA-181b, (d) miRNA-155.

The selectivity of the impedimetric miRNA-34a biosensor platform was tested against other microRNA sequences: miRNA-550 and miRNA-181b. In this respect, hybridization between 1 µg/mL miRNA-34a DNA probe and 10 µg/mL miRNA-34a target, or noncomplementary miRNAs was performed and the average R_ct_ values recorded as 692.71 ± 85.77 Ohm; 512.67 ± 109.50 Ohm; 563.20 ± 107.44 Ohm; with the RSDs% as 12.38%, 21.27%, 19.08% (shown in Figure 6), respectively. The highest R_ct_ value was determined after hybridization between miRNA-34a specific probe and its complementary miRNA-34a RNA target.

The hybridization between 1 µg/mL miRNA 34a DNA probe and miRNA-34a target, or non-complementary miRNAs; miRNA15a, and miRNA-660 was also performed in fetal bovine serum (FBS) medium. Firstly, the dilution ratio of FBS was investigated based on the sensor response. Different dilution ratios of FBS:PBS (1:1, 1:5, 1:10, 1:20) were used and accordingly, the impedimetric measurements were performed (Figure S7). Since low R_ct_ value with good reproducibility was obtained in the medium of 1:10 (FBS:PBS), it was chosen as the optimum dilution ratio for further experiments.

The hybridization between miRNA-34a DNA probe and miRNA-34a RNA target was performed in FBS medium in the presence of different miRNA-34a target concentrations ranging from 2 to 12 µg/mL. The R_ct_ value was increased till 10 µg/mL miRNA-34a target (Figure S8B) then it leveled off (Figure S8A–g). The detection limit (DL) [33] was estimated in the linear concentration range from 2 to 10 µg/mL (Figure S8B) and found to be 0.826 µg/mL (4.68 pM in 40 μL sample) with a regression equation y = 39.826x − 22.071 with the coefficient of determination (R^2^) of R² = 0.992.

**Figure 7:**
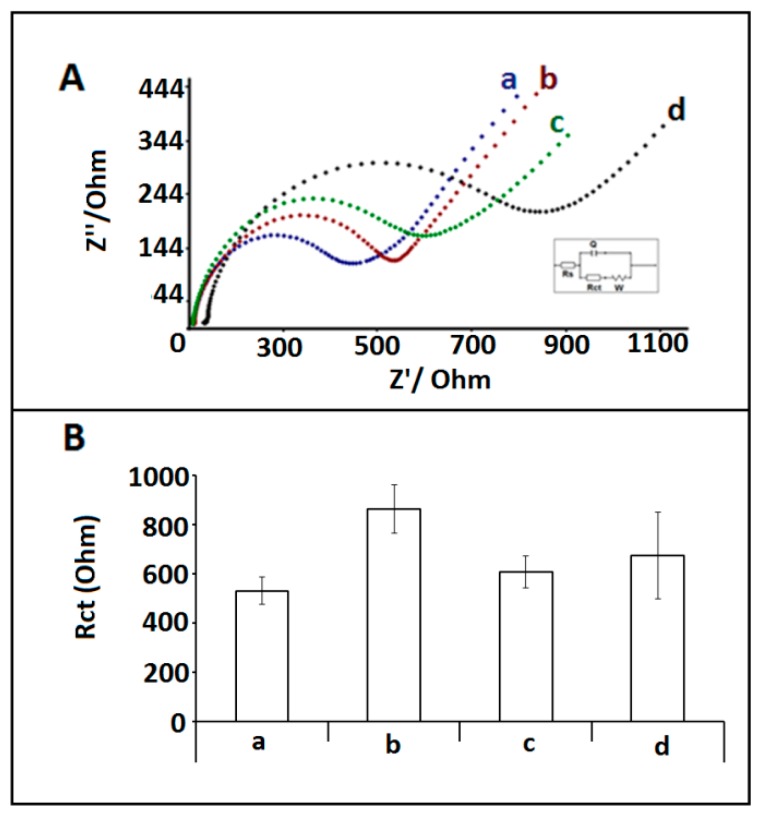
(**A**) Nyquist diagrams representing the R_ct_ values obtained by (**a**) 1 µg/mL miRNA-34a probe modified /IL/CA/PGE in FBS (1:10), hybridization between 1 µg/mL miRNA-34a DNA probe and 10 µg/mL (**b**) miRNA-34a target, (**c**) miRNA-155a, (**d**) miRNA-181b in FBS (1:10). (**B**) Histograms representing the R_ct_ values obtained by (**a**) 1 µg/mL miRNA-34a probe modified /IL/CA/PGE in FBS (1:10), after hybridization between 1 µg/mL miRNA-34a DNA probe and 10 µg/mL (**b**) miRNA-34a target, (**c**) miRNA-155, (**d**) miRNA-181b in FBS (1:10).

Next, the selectivity of the impedimetric biosensor was tested against miRNA 155 and miRNA 181b in the presence of 1:10 FBS: PBS diluted solution the changes at the R_ct_ were accordingly calculated in the case of hybridization in diluted FBS, and the results were compared to the one obtained by pseudo hybridization using miRNA-34a DNA probe (Figure 7). The average R_ct_ values were recorded as 861.43 ± 98.60, 605.97 ± 65.36, and 673.25 ± 174.34 Ohm with the RSDs% 11.45%, 10.79%, and 25.90% (n = 3) after the hybridization of DNA probe and miRNA-34a, miRNA-155, and miRNA-181b, respectively. 62.4%, increase R_ct_ value were obtained after the hybridization of DNA probe and miRNA-34a. On the other hand, 14.20% and 27.91% increase were calculated respectively at the R_ct_ value in the case of hybridization with miRNA-155 and miRNA-181b. 

It could be concluded that our impedimetric biosensor based on IL-modified covalently activated disposable PGEs showed a selective behavior to specific miRNA-34a detection even in artificial serum medium due to the highest increase ratio at R_ct_ was recorded after full hybridization of DNA probe with miRNA-34a target.

## 4. Conclusions

Ionic liquid-modified chemically activated graphite electrodes were developed for the first time in the literature, and they were applied for impedimetric detection of miRNA-34a. The characterization of IL/CA/PGEs was performed using not only electrochemical techniques, but also by SEM. Under the optimized experimental conditions, our impedimetric miRNA-34a biosensor presented a good hybridization selectivity against other miRNAs (miRNA-155, and miRNA-181b) even if more than seven bases of these miRNAs were the same with the miRNA-34a target base sequence. There have been many studies [31,34,35,36,37,38,39,40] presenting the detection of miRNA using different types of electrochemical transducers/electrodes, and some of these studies were summarized in Table 1. In comparison to the previous reports, our proposed electrochemical biosensor platform presented some advantages; such as, easy-to-handle, cost-effective, fast, disposable electrode surface modification and portable. In this aspect, our biosensor provides a cost-effective assay in contrast to the earlier studies performed using screen-printed electrodes [41], or gold electrodes [42], AuNPs-modified electrodes [43] and some optical biosensor systems [44]. Additionally, the detection of miRNA achieved in both buffer (PBS) and diluted FBS medium within detection limits of 109 nM and 117 nM, respectively.

Ionic liquid-modified chemically activated PGEs offer advanced detection methodologies for the detection of nucleic acids. The IL-based disposable sensor platform developed in our study presents great promise for development of low-cost and sensitive biosensing protocol for healthcare monitoring as well as environmental analysis.

## Figures and Tables

**Table 1:** Recent studies developed for miRNA detection compared to the present study. Abbreviations: Electrodes: PGE: pencil graphite electrode, SPE: screen printed electrode, GCE: glassy carbon electrode, AuE: gold electrode. Modification materials: GO: graphene oxide, CNF: carbon nanofiber AuNPs: gold nanoparticles, MgO: magnesium oxide, MoS_2_: molybdenum disulfide, IL: ionic liquid, LNA/MB: locked nucleic acid/molecular beacon, CA: chemically activated. Technique: SWV: square wave voltammetry, CV: cyclic voltammetry, DPV: differential pulse voltammetry, EIS: electrochemical impedance spectroscopy.

Electrode	miRNA	Method	DL	Reference
GO/CA/PGE	miRNA-34a	EIS	82.36 nM	[31]
CNFs/SPE	miRNA-34a	DPV, EIS	3.12 µM	[34]
AuNPs/AuE	miRNA let-7d	EIS, CV, SWV,	0.17 pM	[35]
MWCNT modified GCE	miRNA-155	CV, DPV	1.64 fM	[36]
specific biotinylated DNA/LNA/MB/AuNPs/GCE	miRNA-21	EIS	0.3 pM	[37]
AuNPs/MoS_2_/GCE	miRNA-21	CV, DPV, EIS	0.086 fM	[38]
PPy-PGE	miRNA-34a	EIS	0.20 µg/mL	[39]
GO/CA/PGE	miRNA-34a	DPV	1.07 µM	[40]
IL/CA/PGE	miRNA-34a	EIS	0.772 µg/mL (0.109 µM)	This study

## Supplementary Materials

The following document is available online at http://www.mdpi.com/1424-8220/18/9/2868/s1, Figure S1: Histograms representing the average oxidation signal of (a) PGE (b) CA/PGE, %IL modified CA/PGE (c) 5% (d) 10%, Figure S2: (A) Nyquist diagrams (B) Histograms representing the R_ct_ values obtained by (a) PGE, (b) CA/PGE, 20% IL modified CA/PGE, (c) 15 min, (d) 30 min, (e ) 60 min. (e) 15% (f) 20% (g) 25% (h) 30%, Figure S3: Histograms representing the R_ct_ values obtained by (a) PGE, (b) CA/PGE, (c) IL/CA/PGE, 1 μg/mL probe modified (a’) PGE, (b’) CA/PGE, (c’) IL/CA/PGE. Inset was the apparent fractional coverage (Q^IS^_R_) values of 1μg/mL miRNA-34a DNA probe modified (a) PGE, (b) CA/PGE, (c) IL/CA/PGE, Figure S4: (A) Nyquist diagrams representing the R_ct_ values obtained by (a) CA/PGE, (b) IL/CA/PGE, (c) Hybrid between 1 μg/mL miRNA-34a probe and 10 μg/mL target modified IL/CA/PGE. (B) Histograms representing the R_ct_ values obtained by (a) CA/PGE, (b) IL/CA/PGE, (c) Hybrid between 1 μg/mL miRNA-34a probe (d) 0.5 μg/mL probe (e) 1.5 μg/mL probe and 10 μg/mL target modified IL/CA/PGE, Figure S5: Histograms representing the R_ct_ values obtained by (a) PGE, (b) CA/PGE, 20% IL modified CA/PGE, pseudo hybridization of 1 μg/mL miRNA-34a DNA probe immobilization onto the surface of IL/CA/PGE in various time (d) 5 min, (e ) 15 min, (f) 30 min, (g) 45 min, after hybridization between 1 μg/mL miRNA-34a DNA probe and 10 μg/mL miRNA-34a target immobilization onto the IL/CA/PGE surface in various time (d’) 5 min, (e’) 15 min, (f’) 30 min, (g’) 45 min, Figure S6: Histograms representing the R_ct_ values obtained by (a) PGE, (b) CA/PGE, 20% IL modified CA/PGE, pseudo hybridization of 1 μg/mL miRNA-34a DNA probe onto the surface of IL/CA/PGE in different time (d) 5 min, (e ) 15 min, (f) 30 min, after hybridization between 1 μg/mL miRNA-34a DNA probe and 10 μg/mL miRNA-34a target immobilization onto the IL/CA/PGE surface in different hybridization time (d’) 5 min, (e’) 15 min, (f’) 30 min, Figure S7: Histograms representing the R_ct_ values obtained by (a) PGE, (b) CA/PGE, 20% IL modified CA/PGE, pseudo hybridization of 1 μg/mL miRNA-34a DNA probe onto the surface of IL/CA/PGE in different FBS dilution rate (d) 1:1, (e), 1:5, (f) 1:10, (g) 1:20, Figure S8: Histograms representing the R_ct_ values obtained by (a) pseudo hybridization of 1 μg/mL miRNA-34a DNA probe in diluted FBS (1:10), after hybridization between 1 μg/mL miRNA-34a 59 DNA probe and different miRNA-34a target concentrations (b) 2 μ/mL, (c) 4 μg/mL, (d) 6 μg/mL (e) 8 μg/mL, (f) 10 μg/mL (g) 12 μg/mL. (B) The calibration graph obtained from R_ct_ values measured in the presence of different concentration of miRNA-34a target, ranging from 2–10 μg/mL, Table S1: According to the CV measurements; average anodic peak currents (Ia) average cathodic peak current (Ic), anodic charge values (Qa) and calculated surface areas (A) of PGE, CA/PGE and % IL modified CA/PGEs, 5% to 30%, Table S2: The Q^IS^_R_ values obtained using hybrid immobilized IL/CA/PGE at different concentration level of miRNA-34a DNA target from 2 to 12 μg/mL.
