# Supplementary material for: An Impedimetric Biosensor Based on Ionic Liquid-Modified Graphite Electrodes Developed for microRNA-34a Detection
Source: Sensors (Basel). 2018 Aug 31;18(9):2868. doi: 10.3390/s18092868 (PMC6164345; doi:10.3390/s18092868)
Supplement: Supplementary file 1 [file sensors-18-02868-s001.pdf]

Supplementary Material

Article for special issue "Immunosensors - 2018 Trends and Perspective"

Impedimetric Biosensor Based on Ionic Liquid Modified Graphite Electrodes Developed for miRNA-34a Detection

Ece Kesici [1,2], Ece Eksin[1,2] and Arzum Erdem [1,2,*]

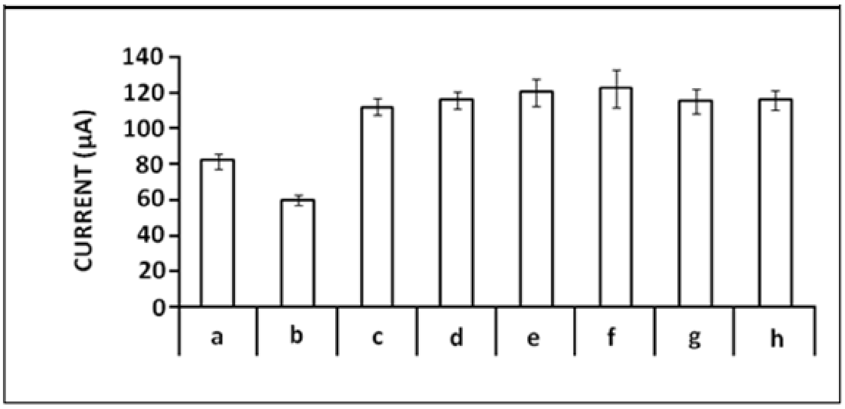

Fig. S1. Histograms representing the anodic peak currents (I_a) of (a) PGE (b) CA/PGE, %IL modified CA/PGE (c) 5% (d) 10% (e) 15% (f) 20% (g) 25% (h) 30%. The CV measurements were conducted in 2 mM $K_4[Fe(CN)_6]$ / $K_3[Fe(CN)_6]$ (1:1) prepared in 0.1 M KCl.

Table S1. According to the CV measurements; average anodic peak currents (I_a) average catodic peak current (I_c), anodic charge values (Q_a) and calculated surface areas (A) of PGE, CA/PGE and % IL modified CA/PGEs, 5% to 30%.

	**I_a (μA)**	**I_c (μA)**	**Q_a**	**Q_c**	**A (cm^2)**	**%**
**PGE**	81.96	88.23	1.15	1.02	0,25 cm^2	-
**CA/PGE**	60.01	73.23	1.08	0.93	0.18 cm^2	-
**5% IL/CA/PGE**	112.10	101.23	1.39	1.29	0.34 cm^2	36.99
**10% IL/CA/PGE**	116.10	103.13	1.39	1.13	0.35 cm^2	42.28

**15% IL/CA/PGE**	120.50	104.16	1.37	1.27	0.36 cm^2	47.97
**20% IL/CA/PGE**	122.33	106.98	1.43	1.30	0.37 cm^2	50.00
**25% IL/CA/PGE**	115.30	105.40	1.34	1.23	0.35 cm^2	41.05
**30% IL/CA/PGE**	116.23	99.14	1.36	1.23	0.36 cm^2	44.31

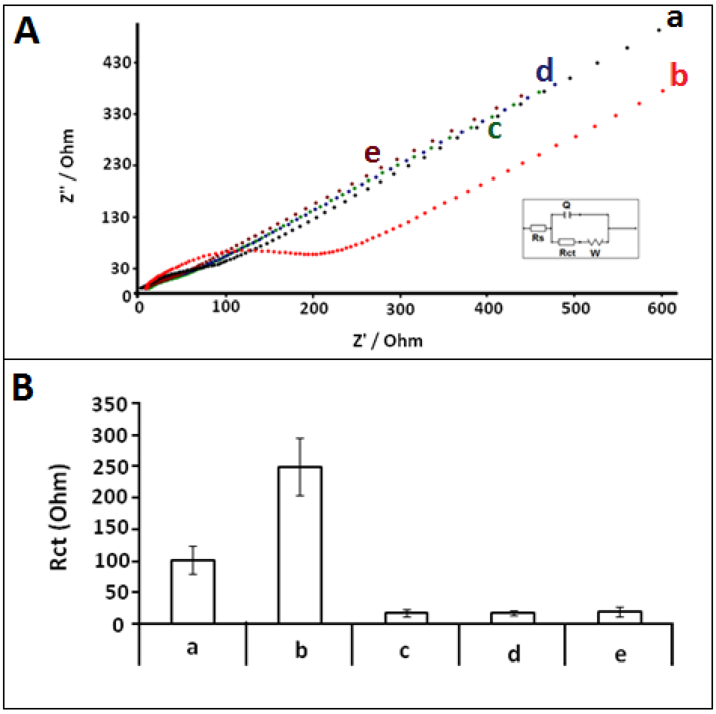

Fig. S2. (A) Nyquist diagrams (B) Histograms representing the R_{ct} values obtained by (a) PGE, (b) CA/PGE, 20% IL modified CA/PGE, (c) 15 min, (d) 30 min, (e) 60 min.

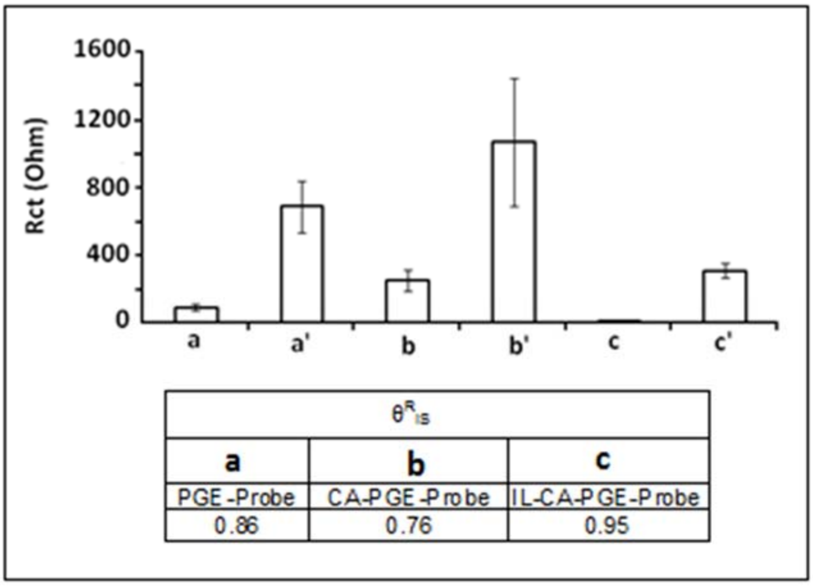

Fig. S3. Histograms representing the R_{ct} values obtained by (a) PGE, (b) CA/PGE, (c) IL/CA/PGE, 1 µg/mL probe modifed (a') PGE, (b') CA/PGE, (c') IL/CA/PGE. Inset was the appearent fractional coverage (Q^{IS}_{R}) values of 1µg/mL miRNA-34a DNA probe modified (a) PGE, (b) CA/PGE, (c) IL/CA/PGE.

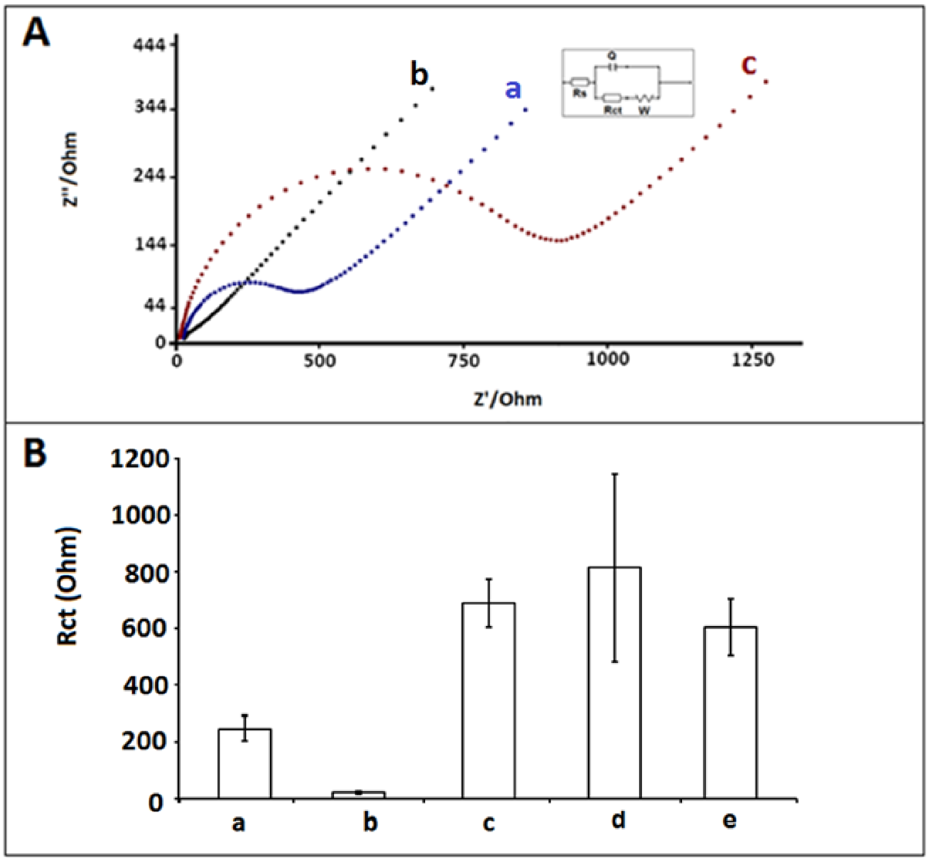

Fig. S4. (A) Nyquist diagrams representing the R_{ct} values obtained by (a) CA/PGE, (b) IL/CA/PGE, (c) Hybrid between 1µg/mL miRNA-34a probe and 10 µg/mL target modified IL/CA/PGE. (B) Histograms representing the R_{ct} values obtained by (a) CA/PGE, (b) IL/CA/PGE, (c) Hybrid between 1µg/mL miRNA-34a probe (d) 0.5 µg/mL probe (e) 1.5 µg/mL probe and 10 µg/mL target modified IL/CA/PGE.

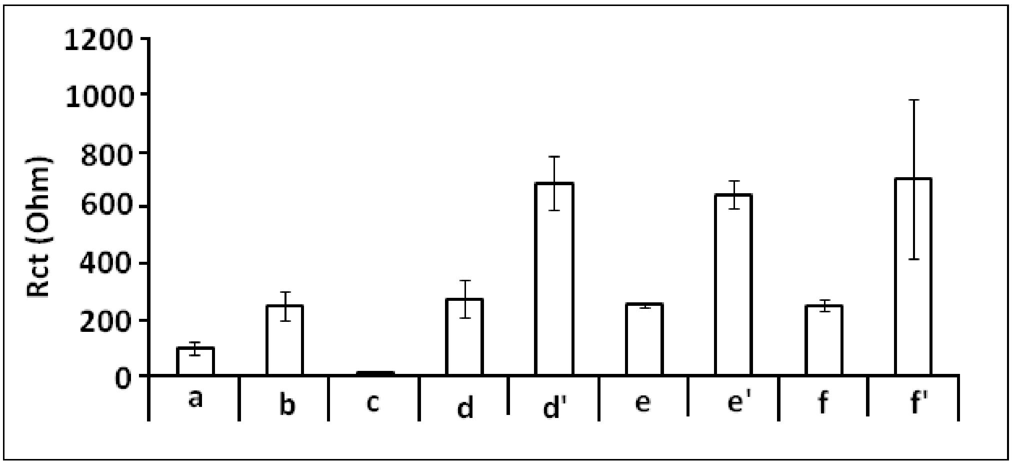

Fig. S5. Histograms representing the R_{ct} values obtained by (a) PGE, (b) CA/PGE, 20% IL modified CA/PGE, pseudo hybridization of 1 µg/mL miRNA-34a DNA probe onto the surface of IL/CA/PGE in different time (d) 5 min, (e) 15 min, (f) 30 min, after hybridization between 1 µg/mL miRNA-34a DNA probe and 10 µg/mL miRNA-34a target immobilization onto the IL/CA/PGE surface in different hybridization time (d') 5 min, (e') 15 min, (f') 30 min.

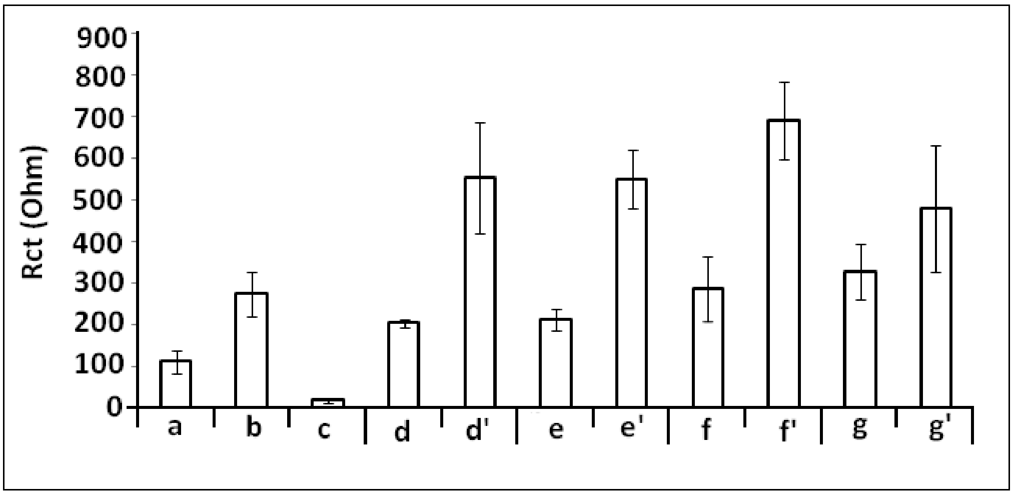

Fig. S6. Histograms representing the R_{ct} values obtained by (a) PGE, (b) CA/PGE, 20% IL modified CA/PGE, pseudo hybridization of 1 µg/mL miRNA-34a DNA probe immobilization onto the surface of IL/CA/PGE in various time (d) 5 min, (e) 15 min, (f) 30 min, (g) 45 min, after hybridization between 1 µg/mL miRNA-34a DNA probe and 10 µg/mL miRNA-34a target immobilization onto the IL/CA/PGE surface in various time (d') 5 min, (e') 15 min, (f') 30 min, (g') 45 min.

Table S2. The Q^{IS}_{R} values obtained using hybrid immobilized IL/CA/PGE at different concentration level of miRNA-34a DNA target from 2 to 12 µg/mL.

miRNA-34a target concentration (µg/mL)	PGE-CA-IL-hybrid Q^{IS}_{R}
**2**	0.823
**4**	0.906
**6**	0.928
**8**	0.942
**10**	0.955
**12**	0.919

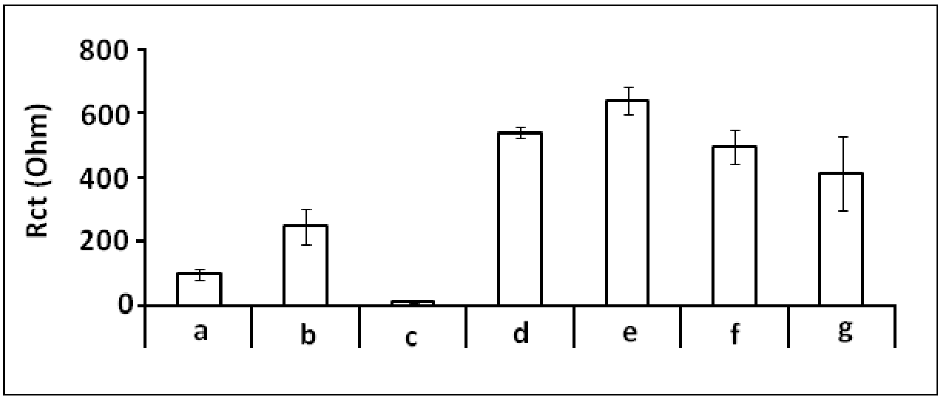

Fig. S7. Histograms representing the R_{ct} values obtained by (a) PGE, (b) CA/PGE, 20% IL modified CA/PGE, pseudo hybridization of 1 µg/mL miRNA-34a DNA probe onto the surface of IL/CA/PGE in different FBS dilution rate (d) 1:1, (e), 1:5, (f) 1:10, (g) 1:20.

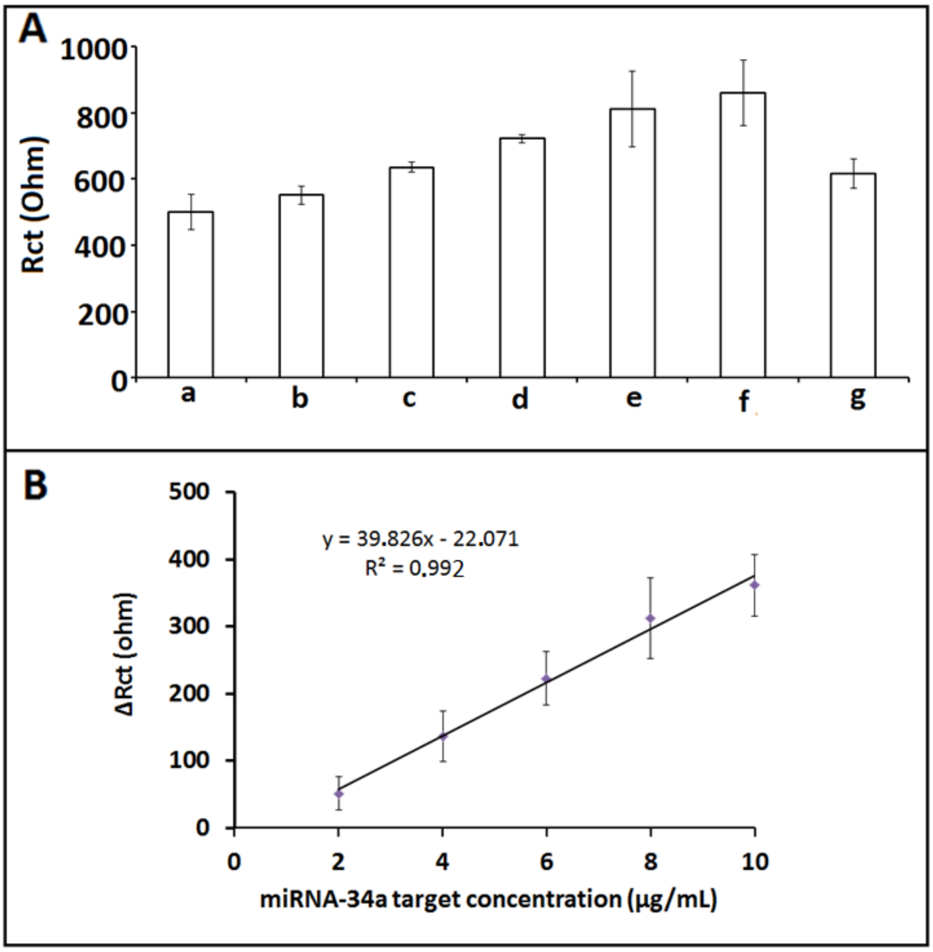

Fig. S8.(A) Histograms representing the R_{ct} values obtained by ~~(a~~) pseudo hybridization of 1 µg/mL miRNA-34a DNA probe in diluted FBS (1:10), after hybridization between 1 µg/mL miRNA-34a DNA probe and different miRNA-34a target concentrations (b) 2 µ/mL, (c) 4 µg/mL, (d) 6 µg/mL (e) 8 µg/mL, (f) 10 µg/mL (g) 12 µg/mL. **(B)** The calibration graph obtained from R_{ct} values measured in the presence of different concentration of miRNA-34a target, ranging from 2-10 µg/mL.
